# Mortality from aluminum phosphide poisoning in Kermanshah Province, Iran: characteristics and predictive factors

**DOI:** 10.4178/epih.e2018022

**Published:** 2018-05-27

**Authors:** Seyed Mohammad Navabi, Jafar Navabi, Abbas Aghaei, Zahra Shaahmadi, Ruhollah Heydari

**Affiliations:** 1 School of Pharmacy, Kermanshah University of Medical Sciences, Kermanshah, Iran; 2 Clinical Research Development Center, Imam Khomeini and Mohammad Kermanshahi Hospitals, Kermanshah University of Medical Sciences, Kermanshah, Iran; 3 School of Medicine, Kermanshah University of Medical Sciences, Kermanshah, Iran; 4 Department of Epidemiology, School of Public Health, Kermanshah University of Medical Sciences, Kermanshah, Iran

**Keywords:** Aluminum phosphide, Rice tablets, Poisoning, Pesticide, Mortality, Iran

## Abstract

**OBJECTIVES:**

Aluminum phosphide (ALP), also known in Iran as rice tablets, is one of the most effective rodenticides used to protect stored grain. However, ALP poisoning regularly causes mortality in humans. The aim of this study was to evaluate the characteristics and predictive factors of mortality from ALP poisoning.

**METHODS:**

This study evaluated all patients with ALP poisoning referred to Imam Khomeini Hospital in Kermanshah Province, Iran from 2014 to 2015. For each patient, the following information was recorded: age, sex, the number of tablets consumed, the number of suicide attempts, the time elapsed from consumption to treatment, blood pressure, blood pH, HCO_3_ levels, and PCO_2_ . Differences between the survivors and non-survivors of ALP poisoning were analyzed using univariate logistic regression and multivariate analysis.

**RESULTS:**

In this study, 48 patients were male and 29 patients were female (total: 77 patients). The average age of the survivors and non-survivors was 28.7 and 31.3 years, respectively. All cases (100%) of ALP poisoning were intentional, with the goal of committing suicide. The main predictive variables of mortality from ALP poisoning were blood pressure, blood pH, and time elapsed from consumption to treatment.

**CONCLUSIONS:**

The likelihood of mortality in patients with ALP poisoning can be predicted using blood pressure, blood pH, and time elapsed from consumption to treatment. These findings may help healthcare providers take more effective measures to treat patients with ALP poisoning.

## INTRODUCTION

Poisoning is one of the main causes of disability and death throughout the world. Poisoning has been reported to account for 1.0-2.0 and 1.3% of deaths in developed countries and Iran, respectively [1,2]. Aluminum phosphide (ALP) is a highly dangerous pesticide that is popularly referred to in Iran as rice tablets [3,4]. ALP is a common cause of acute poisoning and death in many developing countries, especially in Asia, and it usually has high fatality rates (60-90%) [5-11]. This pesticide was first introduced in India, where roughly 15,000 accidental or intentional poisonings take place annually, with a reported mortality rate of roughly 67% [12]. Many studies have also shown the mortality rate from ALP poisoning in Iran to be high [2,11,13,14]. ALP is toxic because it produces phosphine gas in the presence of moisture in the air or water and in the presence of hydrochloric acid in the stomach [3]. The produced phosphine is quickly absorbed through the digestive system when ALP is consumed orally. Therefore, if timely treatment measures are not applied, there is a risk of death [15]. The median lethal dose of ALP for an adult weighing 70 kg is approximately 500 mg [16].

The symptoms of poisoning start within a few minutes after ingestion, after phosphine gas is released through the contact of the ALP tablet with stomach acid. ALP poisoning has several distinct symptoms, including vomiting, abdominal pain, agitation, tachycardia, tachypnea, acidosis, and hypotension. The clinical course of ALP poisoning starts with nausea and vomiting and proceeds to multi-organ failure and death within 24 to 48 hours after poisoning [12,17]. The most common symptoms of severe poisoning are hypotension, shock, and myocardial injuries causing cardiac arrhythmias and conduction disturbances [18].

ALP poisoning most commonly occurs intentionally, with the goal of committing suicide, although some accidental cases of ALP poisoning occur through occupational exposure, and in rare cases, ALP poisoning is committed with criminal intentions. Most poisoning cases occur in young adults (20-30 years). Various factors affect the extent of ALP poisoning, including social, geographical, and psychological status and the availability of ALP [19].

In studies that compared poisoning in different parts of Iran, it has been shown that poisoning with ALP accounted for between 25 and 45% of all poisoning cases [11,20]. In a study conducted by Kordrostami et al. [21] on mortality data from poisonings between 2011 and 2015 in Tehran, it was found that 619 deaths were due to ALP poisoning, accounting for 81% of the total of 764 deaths due to poisoning during those 5 years. These findings confirm the high rates of ALP poisoning and deaths in Iran.

There is a high risk of death from ALP poisoning in Kermanshah Province because of its farmland and abundant grain production, which imply that rice tablets are abundantly available. Factors predicting mortality from ALP poisoning have not been investigated in any studies, and our hypothesis was that some factors, such as blood pH and blood pressure, could predict mortality in patients with ALP poisoning. Therefore, it was necessary to investigate the characteristics and predictive factors of mortality from ALP poisoning.

## MATERIALS AND METHODS

This cross-sectional study evaluated patients with ALP poisoning who were referred to Imam Khomeini Hospital in Kermanshah, Iran from 2014 to 2015. All patients in this time interval were included in the study. Imam Khomeini Hospital is the only referral center for cases of poisoning in western Iran. This hospital is a governmental center that collaborates with all types of insurance programs and offers identical services to all patients without consideration of their socioeconomic status. Information was obtained from the patient (or his or her caregiver) about the patient’s age, sex, the number of tablets consumed, the number of suicide attempts, and the time elapsed from consumption to treatment. Blood pressure, serum HCO_3_ levels, blood pH, and PCO_2_ at admission and after full recovery (or death) were gathered using a checklist. The patients at this center received the standard treatment for ALP poisoning applied by most medical centers in Iran (Table 1), including gastric lavage with potassium permanganate, administration of oral coconut oil, and intravenous administration of sodium bicarbonate, magnesium sulfate, corticosteroids, hydrogen pump blockers, vasopressors, and fluid therapy.

The data were categorized as quantitative or qualitative. Patients were classified according to systolic blood pressure (SBP). Patients with a SBP of at least 90 mmHg were considered not to be in shock, while those with a SBP less than 90 mmHg were considered to be in shock. Variables with an abnormal and highly skewed distribution were used as qualitative inputs into the logistic regression analysis. In the univariate analysis method, each variable was separately entered into the regression table. In the multivariate analysis method, first, all variables were analyzed in the logistic regression analysis using the backward stepwise and forward stepwise methods, and then the variables with a p< 0.2 from the univariate analysis were included in the multivariate analysis using those 2 methods. SPSS version 16 (SPSS Inc., Chicago, IL, USA) was used for the statistical analysis.

This study was received permission from the ethics committee of the university (approval no. KUMS.RES.1394.481).

## RESULTS

Seventy-seven patients had ALP poisoning. The average age of the survivors and non-survivors was 28.7 and 31.3 years, respectively. The oldest and youngest cases of poisoning were 68.0 and 16.0 years old, respectively. Twenty-nine patients (37.7%) were female and 48 (62.3%) were male. All cases of ALP poisoning (100.0%) were suicide attempts (intentional poisoning). Forty-one patients were not in shock, while 36 patients were in shock. The majority of patients (61.0%) had vomited.

The average values of the studied variables for the survivors and non-survivors were as follows: the number of tablets consumed was 1.7 ± 1.0 and 2.6 ± 1.9, the time elapsed from consumption to treatment was 1.3± 0.7 and 2.0± 1.4 hours, the number of suicide attempts was 1.1± 0.3 and 1.2± 0.8 times, the average blood pH was 7.4± 0.1 and 7.1± 0.2, the average serum HCO_3_ level was 19.1± 4.8 and 12.0± 3.8 mEq/L, and the average PCO_2_ was 32.8± 8.4 and 33.8± 13.7 mmHg in the survivors and nonsurvivors, respectively. More details are presented in Table 1.

The univariate analysis found that mortality had a statistically significant relationship with clinical signs, the number of tablets consumed, having vomited, and serum HCO_3_ (p< 0.05). Additionally, the variable of time elapsed from consumption to treatment had a p< 0.2, and was therefore also entered into the multivariate analysis. More details are presented in Table 2.

In the multivariate logistic regression, the backward stepwise and forward stepwise methods led to the same results, both with all variables and when limited to variables with p< 0.2 in the univariate analysis (Table 3).

The multivariate analysis showed that blood pressure, pH, and time elapsed from consumption to treatment were the most important predictive variables of mortality from ALP poisoning. Blood pressure predicted approximately 59.4% of mortality in cases of poisoning. The combination of blood pressure and blood pH predicted 71.5% of mortality. The combination of blood pressure, blood pH, and time elapsed from consumption to treatment predicted almost 77.3% of mortality in cases of ALP poisoning (Table 3).

## DISCUSSION

This cross-sectional study investigated the characteristics and predictive factors of mortality from ALP. All cases of ALP poisoning from 2014 to 2015 were referred to the Imam Khomeini Hospital, which is the only referral center for poisonings in Kermanshah Province, and were included in this study. Blood pressure, blood pH, and time elapsed from consumption to treatment were found to be the most important determinants of death in these individuals.

The aim of this study was to determine the factors predicting mortality from ALP poisoning. The average age of the survivors and non-survivors of ALP poisoning was 28.7 and 31.3 years, respectively. Taramsary et al. [22] similarly showed that 64.7% of cases of poisoning were in the age group of 15-30 years, with an average age of 29 years. In the study of Khodabandeh et al. [23], the highest prevalence of ALP poisoning (48.6%) was reported in the age group of 15-24 years for both males and females, but most of the non-survivors of ALP poisoning (60.0%) were males aged 15-34.

In this study, most of the reported cases of ALP poisoning were in male patients, consistent with the studies of Khodabandeh et al. [23] and Saha et al. [24]. A reason for this is that rice tablets are more readily available to males, because they are more extensively involved in agricultural work than females. All cases (100.0%) of ALP poisoning were intentional and were suicide attempts. Other studies have also found that ALP poisoning most commonly took place as a means of attempting suicide [3,18]. This may reflect a trend for the increasing use of rice tablets to commit suicide in recent years. According to the results of this study and those of Montazer et al. [25] and Etemadi-Aleagha et al. [26], 61.0, 84.6, and 85.0% of the poisoned patients had vomited by the time of admission, respectively. In our study, 46.8% of patients were in shock, and most of them (91.7%) were non-survivors. Rahbar Taramsary et al. [22] found a similar mortality rate of 92.1% in patients with SBP below 90 mmHg, whereas the mortality rate of patients with SBP over 90 mmHg was 14.3%. Taghadosinejad et al. [27] concluded that shock was one of the most important complications of ALP poisoning, with a high mortality rate. The mean number of tablets consumed by survivors and non-survivors was 1.7± 1.0 and 2.6± 1.9, respectively. Khodabandeh et al. [23] reported that the mean number of tablets consumed was 2.0± 0.5 tablets, in all of patients. However, the most common number of tablets consumed was reported to be 1 tablet (in 41.2% of cases) by Rahbar Taramsary et al. [22]. In the present study, the elapsed time from consumption to treatment was 1.3±0.7 and 2.0±1.4 hours for survivors and non-survivors of ALP poisoning, respectively. Rahbar Taramsary et al. [22] reported that this interval was 1 hour for most of the cases of poisoning (30.9%). However, this interval was reported to be between 2 and 4 hours for 50.5% of the patients by Khodabandeh et al. [23]. In the present study, the mean blood pH was 7.7± 0.1 and 7.1± 0.2 mmHg for the survivors and non-survivors of ALP poisoning, respectively. Rahbar Taramsary et al. [22] similarly reported that 64.6 and 3.6% of patients with a blood pH over 7.2 or below 7.2 mmHg survived ALP poisoning, respectively. In this study, the mean HCO_3_ level was 19.1± 4.8 and 12.0± 3.8 mEq/L for survivors and non-survivors, respectively. Mehrpour et al. [28] reported levels of 12.3± 1.1 and 11.2± 0.9 mEq/L for survivors and non-survivors, respectively.

The mortality rate from ALP poisoning showed a statistically significant relationship with variables including blood pressure, the number of tablets consumed, having vomited, the pH level, the HCO_3_ level (p< 0.05), and time elapsed from consumption to treatment (p< 0.2) based on univariate logistic regression. Multivariate logistic regression showed that blood pressure alone could predict roughly 60% of mortality in the poisoned patients. Furthermore, 71.5 and 77.3% of mortality was predicted by using combinations of 2 (blood pressure and blood pH) and 3 (blood pressure, blood pH, and time elapsed from consumption to treatment) variables, respectively. Similarly, Rahbar Taramsary et al. [22] showed that the survival of patients had a significant relationship with the number of tablets consumed, the time elapsed to reach the first treatment center, hypotension, blood pH, and HCO_3_ levels. They showed that the mortality rate of ALP poisoning was higher in patients with a blood pressure below 90 mmHg, a blood pH < 7.2, or an HCO_3_ level<15.0, who took over half of a rice tablet, or for whom more than half an hour elapsed from consumption to treatment.

The results of the multivariate analysis indicated that the likelihood of dying increased by about 59 and 12 times if patient was in shock or had experienced an elapsed time of more than 1 hour to reach treatment after ALP poisoning, respectively. Moreover, the likelihood of dying decreased by about two times with each unit (1 mmHg) increase in blood pH. Therefore, accelerating the transport of poisoned individuals to treatment centers, followed by careful attention to blood pressure and blood pH at the time of treatment, would be expected to reduce the risk of mortality in cases of ALP poisoning.

One of the strengths of this study is that all cases of ALP in Kermanshah Province during the study period were evaluated, meaning that this study has good generalizability. Furthermore, appropriate analytical methods were used in this study, unlike similar studies in this field.

The main limitation of this study is that some of the variables were measured indirectly (reported by the patients’ companions or clinical caregivers), but since this issue occurred to the same extent for all cases, any resulting information bias is expected to be non-differential regarding outcomes.

The results of this study showed that mortality can be predicted in patients with ALP poisoning based on their blood pressure, blood pH, and time elapsed from consumption to treatment. These findings may help healthcare providers take more effective measures to treat patients with ALP poisoning.

## Figures and Tables

**Table 1.** Demographic and clinical characteristics of patients with ALP poisoning in Kermanshah Province, 2014-2015

Variables		Total (n=77)	Survivors	Non-survivors	Min	Max	Percentile		
							25th	50th	75th
Qualitative									
Blood pressure^1^	Non-shock	41 (53.2)	33 (80.5)	8 (19.5)	-	-	-	-	-
	Shock	36 (47.8)	3 (8.3)	33 (91.7)	-	-	-	-	-
Sex	Female	29 (37.7)	15 (51.7)	14 (48.3)	-	-	-	-	-
	Male	48 (62.3)	21 (43.8)	27 (56.3)	-	-	-	-	-
Vomiting	No	30 (39.0)	3 (10.0)	27 (90.0)	-	-	-	-	-
	Yes	47 (61.0)	33 (70.2)	14 (29.8)	-	-	-	-	-
Quantitative									
Age (yr)			28.7±10.2	31.3±10.7	16.0	68.0	23.0	27.0	35.0
No. of tablets consumed			1.7±1.0	2.6±1.9	1.0	10.0	1.0	2.0	3.0
Time elapsed from consumption to treatment (hr)			1.3±0.7	2.0±1.4	0.5	7.0	1.0	1.0	2.0
Suicide attempt(s)			1.1±0.3	1.2±0.8	1.0	6.0	1.0	1.0	1.0
Blood pH			7.4±0.1	7.1±0.2	6.4	7.5	7.1	7.3	7.4
HCO_3_ (mEq/L)			19.1±4.8	12.0±3.8	4.6	25.8	12.0	15.0	20.1
PCO_2_ (mmHg)			32.8±8.4	33.8±13.7	13.5	76.0	25.5	33.5	38.8

Values are presented as number (%) or mean±standard deviation. ALP, aluminum phosphide; Min, minimum; Max, maximum.

1 Non-shock: cases with systolic blood pressure over (or equal to) 90 mmHg; Shock: cases with systolic blood pressure below 90 mmHg.

**Table 2.** Univariate logistic regression analysis for factors related to mortality from ALP poisoning in Kermanshah Province, 2014-2015

Variables		n (%)	β	OR (95% CI)	p-value
Blood pressure^1^	Non-shock^1^	41 (53.2)	-	1.00 (reference)	
	Shock	36 (46.8)	3.82	45.37 (11.06, 186.21)	<0.001
Sex	Female	29 (37.7)	-	1.00 (reference)	
	Male	48 (62.3)	0.32	1.38 (0.55, 3.47)	0.50
No. of tablets consumed	1	24 (31.2)	-	1.00 (reference)	
	2	18 (23.4)	0.98	2.66 (0.83, 8.57)	0.10
	>2	35 (45.5)	1.41	4.11 (1.35, 12.54)	0.01
Time elapsed from consumption to treatment (hr)	≤1	33 (42.9)	-	1.00 (reference)	
	>1	44 (57.1)	0.74	2.10 (0.83, 5.30)	0.12
Suicide attempt(s)	1	71 (92.2)	-	1.00 (reference)	
	>1	6 (7.8)	-0.14	0.87 (0.16, 4.60)	0.87
Vomiting	No	30 (39.0)	-	1.00 (reference)	
	Yes	47 (61.0)	-3.06	0.05 (0.01, 0.18)	<0.001
Age		77 (100.0)	0.03	1.03 (0.98, 1.08)	0.27
Blood pH		77 (100.0)	-0.98	0.38 (0.24, 0.59)	<0.001
HCO_3_		77 (100.0)	-0.37	0.69 (0.59, 0.81)	<0.001
PCO_2_		77 (100.0)	0.01	1.01(0.97, 1.05)	0.70

ALP, aluminum phosphide; OR, odds ratio; CI, confidence interval.

1 Non-shock: cases with systolic blood pressure over (or equal to) 90 mmHg; Shock: cases with systolic blood pressure below 90 mmHg.

**Table 3.** Multivariate logistic regression analysis for factors related to mortality and their predictive power in patients with ALP poisoning in Kermanshah Province, 2014-2015

Variables		n (%)	β	OR (95% CI)	p- value	Nagelkerke R^2^		
Blood pressure (1)^1^	Non-shock	41(53.2)	-	1.00 (reference)		(1)		
	Shock	36 (46.8)	4.07	58.69 (6.26, 549.97)	<0.001	0.594		
Blood pH (2)		77 (100.0)	-0.67	0.51 (0.30, 0.87)	0.01		(1) & (2)	
							0.715	
Time elapsed from consumption to treatment, hr (3)	≤1	33 (42.9)	-	1.00 (reference)				(1) & (2) & (3)
	>1	44 (57.1)	2.52	12.39 (1.34, 114.70)	0.03			0.773

ALP, aluminum phosphide; OR, odds ratio; CI, confidence interval.

1 Non-shock: cases with systolic blood pressure over (or equal to) 90 mmHg; Shock: cases with systolic blood pressure below 90 mmHg.
